# Evaluation of Ocular Irritation Sensitivity: Implications of Clinical Parameters, Pain Sensitivity, and Tear Neuromediator Profiles

**DOI:** 10.3390/jcm14010138

**Published:** 2024-12-29

**Authors:** Hyeon-Jeong Yoon, Ja Young Moon, Hyun Jee Kim, Sodam Park, Ji Suk Choi, Hoon-In Choi, Seoyoung Kim, Kyung Chul Yoon

**Affiliations:** 1 Department of Ophthalmology, Chonnam National University Medical School and Hospital, Gwangju 61469, Republic of Korea; yoonhyeonjeong@daum.net (H.-J.Y.);; 2 Safety and Microbiology Lab, Amorepacific R&I Center, Yongin-si 17074, Republic of Korea

**Keywords:** ocular irritation sensitivity, tear neuromediators, pain sensitivity, cosmetic safety assessment, calcitonin gene-related peptide, substance P

## Abstract

**Background/Objectives:** Sensitivity to ocular irritation varies among individuals, being influenced by clinical, subjective, and biochemical factors. This study aimed to evaluate individual variability in ocular irritation sensitivity, focusing on clinical parameters, pain perception, and tear neuromediator profiles. **Methods:** Sixty female participants aged 20–40 were classified into high-sensitivity and low-sensitivity groups based on their response to an irritant (Tween20). Clinical assessments included the ocular surface disease index (OSDI), tear break-up time (TBUT), Schirmer test, and corneal touch threshold measured with the Cochet–Bonnet esthesiometer. Pain sensitivity was assessed using the pain sensitivity questionnaire (PSQ), and tear neuromediators were quantified in tear samples before and after stimulation. The concentrations of calcitonin gene-related peptide (CGRP), nerve growth factor, neuropeptide Y, vasoactive intestinal peptide (VIP), and substance P were measured using an enzyme-linked immune sorbent assay (ELISA). **Results:** The high-sensitivity group exhibited significantly higher OSDI scores (*p* = 0.038). No significant differences were observed in TBUT, corneal staining scores, or Schirmer’s test results. The PSQ results revealed that the high-sensitivity group had lower total and moderate pain scores (*p* = 0.037 and *p* = 0.040, respectively). An analysis of the tear neuromediator showed elevated baseline CGRP levels (*p* = 0.017) and a significant post-stimulation increase in substance P (*p* = 0.021) in the high-sensitivity group. **Conclusions:** These findings emphasize the value of combining clinical, subjective, and biochemical measures to understand sensitivity to ocular irritation. This comprehensive approach may guide the development of safer cosmetic formulations and improve safety assessment protocols.

## 1. Introduction

The evaluation of eye irritation caused by cosmetic products, particularly those used in sensitive areas such as the eyes, is essential for ensuring consumer safety and comfort [1]. Considering the widespread use of cosmetics, understanding and addressing the potential irritation induced by these formulations during ocular exposure is crucial [1,2]. Accidental contact with these products can result in discomfort and, in some cases, damage to the ocular surface [2,3].

Cosmetic formulations frequently include surfactants, which are indispensable for cleansing and emulsification but are also common triggers of eye irritation [3,4,5]. Surfactants, such as cocamide monoethanolamide and sodium lauryl sulfate, have been shown to activate the TRPV1 receptor, a nociceptive ion channel associated with the sensation of burning and stinging pain in ocular tissues [3,4,5]. This receptor, predominantly expressed in sensory C-fiber neurons, is highly responsive to irritants and has been a central focus of in vitro assays designed to evaluate eye irritation [5,6].

Current in vitro assays, such as the NociOcular test, employ TRPV1-specific models to predict irritation potential by measuring calcium flux induced by test materials [7,8]. This assay underscores the role of TRPV1 as a reliable mechanistic endpoint for evaluating eye-sting potential, particularly in formulations containing known irritants like capsaicin [7]. While these models provide valuable insights, individual variability in pain sensitivity poses challenges for predictions [9]. Some individuals exhibit heightened sensitivity to specific products, while others report minimal irritation [9].

This study aimed to identify the characteristics of individuals susceptible to ocular irritation. Tear neuromediators such as calcitonin gene-related peptides (CGRPs) are known to play critical roles in nociceptive signaling and inflammation, serving as potential biomarkers for ocular irritation sensitivity [10,11,12,13]. Clinical assessments, including ocular surface disease index (OSDI) scores and corneal touch thresholds, provide measurable endpoints for subjective discomfort and physical sensitivity, which are essential for understanding real-world product interactions [14,15]. Additionally, tools such as the pain sensitivity questionnaire (PSQ) facilitate the evaluation of an individual’s general pain perception, helping to contextualize variations in subjective irritation responses [9,16]. By integrating these parameters, this study aimed to enhance the understanding of individual differences in ocular irritation sensitivity and bridge the gaps between physiological mechanisms and subjective experiences.

## 2. Materials and Methods

### 2.1. Subjects

Sixty participants were recruited for this study. Ethical approval was obtained from the Chonnam National University Hospital Institutional Review Board (CNUHIRB-2020-144), and the study protocol adhered to the guidelines of the Declaration of Helsinki. The study included female participants aged 20–40 years as they are the primary users of eye-related cosmetic products. All subjects had no history of ophthalmologic diseases (e.g., ocular surface disorders, allergies, etc.) or recent ocular surgery. Informed consent was obtained from all 60 eligible volunteers who were deemed eligible and enrolled in the study.

### 2.2. Experimental Design and Screening for Ocular Sensitivity

This study was conducted in two phases to accurately identify and classify participants based on their sensitivity to ocular irritation. In the first phase, a screening process was conducted to categorize the participants into sensitivity groups. The eye was artificially stimulated by applying a test substance to the periocular region of the right eye. The test substance, a surfactant (Tween20; reagent grade; AMRESCO Inc., Solon, OH, USA), was diluted to 50% with P/S and soaked into 2.5 × 2.5 cm gauze. All participants gently wiped their eyelids with the gauze to avoid direct exposure to the ocular surface. Following the stimulation, participants were instructed to complete a questionnaire evaluating four predefined symptoms within 10 min: burning, tearing, foreign body sensation, and tingling. During the preliminary test, no adverse effects such as skin reactions were observed, aside from the expected sensation of irritation. Participants were classified into two groups based on their responses: those who reported experiencing at least one of the four symptoms within 10 min were assigned to the high-sensitivity group, while those who reported none of the symptoms were in the low-sensitivity group. Based on the pre-screening results, 30 participants were recruited from each group for the second phase of the study.

### 2.3. Measurement of Ocular Surface Parameters

Clinical parameters were evaluated by the same investigator (H.J.Y.), and only the right eye was assessed.

The OSDI questionnaire was used to quantify vision-related quality of life. It assessed three subscales: (1) ocular symptoms (OSDI symptoms), (2) vision-related activities of daily living (OSDI visual function), and (3) environmental triggers (OSDI triggers). The total OSDI scores, each ranging from 0 to 100, were analyzed [17,18].

Tear break-up time (TBUT) was assessed using a moistened fluorescein strip (Haag-Streit, Koeniz, Switzerland). The interval between the last complete blink and the first appearance of a dry spot or disruption in the tear film was recorded in seconds. The examination was performed three times, and the mean break-up time was calculated for analysis. CSSs were obtained using the Oxford grading scale [19]. The Schirmer test score, representing the length of wetting, was measured using a calibrated sterile strip placed at the lateral canthus for 5 min under 0.5% proparacaine. Corneal sensitivity was evaluated using a Cochet–Bonnet esthesiometer. The filament was extended to its full length of 6 cm and shortened incrementally in 0.5 cm steps until the subject perceived contact. The filament length at detection was recorded.

### 2.4. Evaluation of Pain Sensitivity

Pain sensitivity was assessed using the PSQ, a validated self-report tool that evaluates pain perception through imagined everyday scenarios involving various pain stimuli [9,16]. The PSQ consists of 17 items rated on a 0–10 numeric scale, where 0 represents “not painful at all” and 10 indicates the “most severe pain imaginable”.

The PSQ-total score was calculated as the average rating of 14 items (items 1, 2, 3, 4, 6, 7, 8, 10, 11, 12, 14, 15, 16, and 17), excluding 3 non-painful items. Additionally, the PSQ includes two subscales: the PSQ-minor score, derived as the average rating of seven items (items 3, 6, 7, 10, 11, 12, and 14), and the PSQ-moderate score, calculated as the average of seven different items (items 1, 2, 4, 8, 15, 16, and 17).

### 2.5. Tear Collection and Detection of Neuromediators

Tear samples were carefully collected from the inferior tear meniscus of the right eye using sterile glass capillary tubes (Corning, Inc., Corning, NY, USA) to avoid contamination and contact with the ocular surfaces. Two hundred microliters of tear samples were collected and diluted with phosphate-buffered saline (PBS) at a 1:4 ratio. The samples were stored at −70 °C until further analysis.

A specific enzyme-linked immunosorbent assay (ELISA) was performed to quantify neuromediator concentrations in the tear samples, following the manufacturer’s instructions [20]. Tear samples were centrifuged at 3000× *g* for 10 min. Protein samples (100 μL, 10 μg/mL) of calcitonin gene-related peptide (CGRP; Elabscience, Houston, TX, USA), nerve growth factor (NGF; Elabscience), neuropeptide Y (NPY; Elabscience), vasoactive intestinal peptide (VIP; Elabscience), and substance P (R&D Systems, Minneapolis, MN, USA) were absorbed onto conjugate-coated plates. Neuromediator levels in each sample were measured by comparing their absorption values with a standard curve.

### 2.6. Statistical Analyses

Statistical analyses were performed using the SPSS version 22.0 for Windows (SPSS Inc., Chicago, IL, USA). The normality of the data distribution was assessed using the Shapiro–Wilk test. Data are presented as mean ± standard deviation (SD). An independent *t*-test was used to compare variables between groups. A *p*-value of less than 0.05 was considered statistically significant.

## 3. Results

This study included 60 female participants, with a mean age was 23.4 ± 2.23 years. The baseline clinical parameters of the high- and low-sensitivity groups are summarized in Table 1. No significant differences were found in age, tear film break-up time (TBUT), corneal staining score (CSSs), or Schirmer’s test results between the groups. However, the high-sensitivity group reported significantly higher OSDI scores, specifically for ocular symptoms (*p* = 0.021) and total OSDI scores (*p* = 0.038), indicating a greater subjective perception of ocular irritation. Corneal touch threshold measurements using the Cochet–Bonnet esthesiometer showed abnormal corneal sensitivity in three participants, all of whom were in the high-sensitivity group.

Table 2 summarizes the pain sensitivity questionnaire (PSQ) for both groups. The high-sensitivity group exhibited significantly lower PSQ total scores (*p* = 0.037) and marginally lower PSQ-minor scores (*p* = 0.051) than the low-sensitivity group. Individual items also revealed significant differences. For example, Item 3 (“Imagine your muscles are slightly sore as the result of physical activity”; *p* = 0.024) and Item 14 (“Imagine you shake hands with someone who has a very strong grip”; *p* = 0.032) were rated as less painful by the high-sensitivity group compared to the low-sensitivity group. Similarly, PSQ-moderate scores were significantly lower in the high-sensitivity group (*p* = 0.040). Significant differences were observed on specific items, such as Item 2 (“Imagine you burn your tongue on a very hot drink”; *p* = 0.048) and Item 15 (“Imagine you pick up a hot pot by inadvertently grabbing its equally hot handles”; *p* = 0.048).

**Figure 1:**
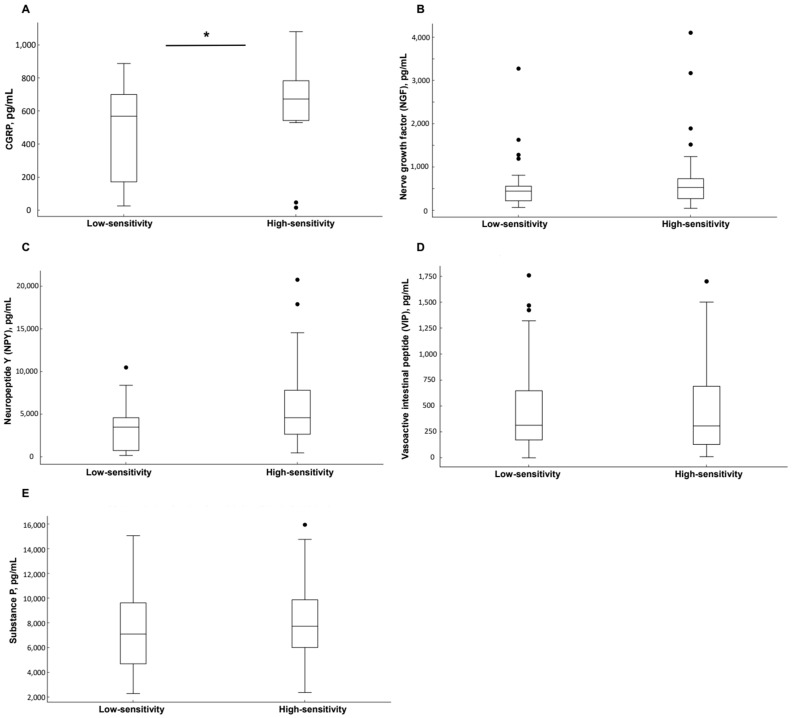
Comparison of tear neuromediators between the sensitivity and no-sensitivity groups of subjects, (**A**) calcitonin gene-related peptide (CGRP), (**B**) nerve growth factor (NGF), (**C**) neuropeptide Y (NPY), (**D**) vasoactive intestinal peptide (VIP), and (**E**) substance P (SP; * *p* < 0.05).

Figure 1 illustrates the differences in baseline tear neuromediator levels between the high- and low-sensitivity groups. The high-sensitivity group demonstrated significantly higher mean levels of CGRP (666.59 ± 274.63 pg/mL) compared to the low-sensitivity group (428.11 ± 280.01 pg/mL; *p* = 0.017). The NGF, NPY, VIP, and substance *p* levels in the high-sensitivity group were 743.21 ± 947.43 pg/mL, 3966.17 ± 2863.53 pg/mL, 552.58 ± 524.42 pg/mL, and 7758.19 ± 3822.45 pg/mL, respectively. In the low-sensitivity group, the corresponding levels were 629.87 ± 625.67 pg/mL, 5838.87 ± 6111.19 pg/mL, 430.01 ± 449.22 pg/mL, and 7366.18 ± 2977.45 pg/mL, respectively. No statistically significant differences were observed for NGF, NPY, VIP, or substance *p* between the two groups.

Table 3 summarizes changes in the tear neuromediator levels before and after stimulation for both groups. In the high-sensitivity group, substance P levels increased significantly after stimulation (*p* = 0.021), suggesting a potential link between substance P and the heightened sensation of ocular irritation in this group. Other neuromediators, including CGRP, NGF, NPY, and VIP, did not show significant changes in either group. In the low-sensitivity group, substance P levels exhibited a non-significant increase post-stimulation (*p* = 0.071).

## 4. Discussion

With the increasing use of cosmetic products applied near the eyes, understanding and managing their potential to cause ocular irritation has become increasingly important [1,2]. Such products require thorough evaluation to ensure they do not cause discomfort, particularly for individuals with heightened sensitivity [1,2,20]. This study aimed to enhance our understanding of individual variability in ocular irritation sensitivity, a critical factor in developing cosmetic products intended for periocular use. Recognizing and addressing this variability is essential for creating products that minimize irritation, especially for sensitive consumers. By identifying specific differences in subjective discomfort perception, physiological sensitivity, and biochemical markers, this study provides valuable insights for targeted safety testing and consumer safety assessments in the cosmetics industry.

The results revealed distinct differences in ocular irritation perception between the high- and low-sensitivity groups, despite minimal variations in most clinical parameters. As shown in Table 1, there were no significant differences between the groups in the TBUT, CSSs, or Schirmer test results. However, the high-sensitivity group reported significantly higher OSDI scores, particularly for ocular symptoms, and total OSDI scores, reflecting a greater subjective perception of discomfort. Interestingly, some participants in the high-sensitivity group exhibited an abnormal corneal touch threshold, suggesting the paradoxical observation that mechanical sensitivity may be reduced in cases of hypersensitivity. One possible explanation for this counterintuitive finding is the presence of central sensitization, in which the central nervous system amplifies the perception of discomfort even in the absence of heightened peripheral sensitivity [21,22]. This discrepancy aligns with prior research, suggesting distinct mechanisms for sensitivity to physical and chemical stimuli [23,24]. For example, individuals may exhibit heightened responses to chemical irritants through central sensitization pathways even when mechanical sensitivity is not elevated [24,25]. These findings underscore the importance of incorporating both subjective and objective measures into product safety testing. Individuals may report high levels of discomfort despite exhibiting low physical sensitivity, emphasizing the need for a multidimensional approach to consumer safety assessments.

The PSQ results (Table 2) further underscored the complexity of discomfort perception in the high-sensitivity group, which scored lower on the PSQ-total and PSQ-moderate than the low-sensitivity group. The PSQ is a validated tool that assesses an individual’s perception of pain intensity in hypothetical scenarios, often reflecting a general tendency toward pain sensitivity rather than situational pain threshold [9,16]. Typically, higher PSQ scores indicate greater sensitivity to imagined pain, whereas lower scores suggest lower sensitivity [9,16]. However, the relationship between PSQ scores and real-time situational discomfort responses remains poorly understood, particularly in the context of ocular irritation [9,26]. Our unexpected result suggests that individuals in the high-sensitivity group may react strongly to immediate real-life discomfort rather than to imagined pain [27]. In other words, their discomfort may be more situational and driven by direct sensory exposure rather than a generalized tendency toward pain sensitivity [27,28]. This finding underscores the importance of including both hypothetical and real-world sensory testing in product trials. Consumers may react differently to actual product exposure than to imagined discomfort. These findings highlight the value of using well-defined sensitivity panels during safety evaluations. By selecting sensitivity panels based on clear and reproducible criteria, developers can accurately predict potential adverse effects that consumers may experience during actual use.

The analysis of tear neuromediators offered additional insights into the physiological mechanisms underlying differences in discomfort sensitivity [20,29,30]. As shown in Figure 1 and Table 3, CGRP levels were elevated at baseline in the high-sensitivity group, and substance P levels increased significantly following stimulation, whereas other neuromediators did not exhibit notable changes. Both CGRP and substance P are closely linked to TRPV1 receptor activation, which mediates nociceptive signaling by modulating neurotransmitter release and transmitting signals through nerve fibers [10,11,12,31,32]. Previous studies have demonstrated an association between elevated CGRP levels and increased pain sensitivity, supporting the hypothesis that high baseline CGRP levels in the high-sensitivity group may contribute to heightened discomfort perception through TRPV1-mediated pathways [12,31,33]. These findings suggest that CGRP and substance P may serve as biomarkers of ocular sensitivity, potentially improving the predictive accuracy of irritation responses during product testing. Monitoring these biomarkers could guide formulation adjustments to reduce discomfort for sensitive users.

As a nonionic surfactant with a low irritation profile, Tween20 is widely used in consumer products and is known to indirectly activate TRPV1 [5,34]. The selection of Tween20 as the test surfactant was based on its suitability as a mild irritant commonly used in cosmetics, with the aim of identifying varying levels of discomfort sensitivity without inducing extreme irritation [35]. By selecting a gentle irritant and diluting it to 50%, this study sought to simulate the typical low-level exposure consumers might experience during daily product use. Despite its mildness, Tween20 successfully differentiated individuals with high sensitivity from those with low sensitivity, confirming its utility as a screening agent for detecting subtle irritation responses relevant to real-world cosmetic formulations.

This study has several limitations. First, the inclusion of only female participants and a relatively narrow age range limit the generalizability of our findings to broader populations, including males and older age groups. Second, while we assessed only the right eye to ensure consistency and minimize discomfort, incorporating the contralateral eye as a control could provide more robust comparative data. Third, although elevated tear neuromediators were observed in the high-sensitivity group, direct assays for TRPV1 activation were not conducted due to limitations in current in vivo methodologies. Future studies addressing these limitations will enhance the understanding of ocular irritation sensitivity and improve the generalizability of these findings.

Nevertheless, the study’s strengths lie in its innovative integration of clinical, subjective, and biochemical parameters, offering a holistic approach to understanding ocular irritation sensitivity. By focusing on female participants, who represent the primary consumers of eye-related cosmetic products, this study provides insights relevant to scenarios of product usage in the real world. To the best of our knowledge, this is the first study to comprehensively evaluate individual variability in ocular irritation. In conclusion, these findings underscore the complex interplay between the subjective and physiological responses to ocular irritation. Recognizing individual differences through a combination of sensory, subjective, and biochemical assessments can enhance product safety testing and ensure a better alignment between product formulations and consumer comfort needs. Future studies should build upon these findings by including more diverse samples in order to further refine sensitivity markers, ultimately supporting the development of safer and more consumer-friendly products.

## Figures and Tables

**Table 1:** Comparison of baseline clinical parameters of subjects in the high-sensitivity and low-sensitivity groups.

	High-Sensitivity Group (N = 30)	Low-Sensitivity Group (N = 30)	*p*-Value
Age, year	23.53 ± 2.08	23.33 ± 2.33	0.99
Clinical parameters			
Tear film break-up time, s	8.33 ± 2.62	8.30 ± 2.42	0.864
Corneal staining score	0.13 ± 0.43	0.17 ± 0.53	0.966
Schirmer test, mm	11.77 ± 5.70	14.27 ± 6.95	0.209
Ocular surface disease index (OSDI)			
OSDI—ocular symptoms	5.49 ± 4.83	3.40 ± 5.14	0.021
OSDI—Vision-related function	3.47 ± 4.45	2.78 ± 5.55	0.086
OSDI—Environmental triggers	4.72 ± 4.43	3.82 ± 4.34	0.211
Total OSDI score	13.68 ± 11.02	10.00 ± 13.75	0.038
Corneal sensitivity (Cochet–Bonnet esthesiometer)			
Corneal touch threshold, mm	59.3 ± 2.13	60.0	0.049
Abnormal (≤55 mm), n	3 (10.0%)	0 (0.0%)	0.119

Data are expressed as mean ± standard deviation.

**Table 2:** Comparison of pain sensitivity questionnaire (PSQ) of subjects in the high-sensitivity and low-sensitivity groups.

	High-Sensitivity Group (N = 30)	Low-Sensitivity Group (N = 30)	*p*-Value
PSQ total	3.93 ± 1.24	4.51 ± 1.50	0.037
PSQ-minor	3.17 ± 1.28	3.68 ± 1.44	0.051
Item 3	3.27 ± 1.44	4.30 ± 1.92	0.024
Item 14	2.13 ± 1.69	3.13 ± 2.00	0.032
PSQ-moderate	4.69 ± 1.32	5.33 ± 1.65	0.040
Item 2	4.20 ± 1.51	5.27 ± 2.11	0.048
Item 15	5.10 ± 1.97	6.10 ± 1.81	0.048

Data are expressed as mean ± standard deviation. Item 3, “Imagine your muscles are slightly sore as the result of physical activity”; Item 14, “Imagine you shake hands with someone who has a very strong grip”; Item 2, “Imagine you burn your tongue on a very hot drink”; Item 15, “Imagine you pick up a hot pot by inadvertently grabbing its equally hot handles”.

**Table 3:** Changes in the levels of tear neuromediators before and after stimulation in the high-sensitivity and low-sensitivity groups.

	Before Stimulation	After Stimulation	*p*-Value
High-sensitivity group (pg/mL)			
CGRP	551.93 ± 196.20	656.48 ± 309.52	0.170
NGF	629.87 ± 625.67	600.64 ± 543.71	0.843
NPY	4964.82 ± 5443.41	5613.82 ± 5200.34	0.613
VIP	445.92 ± 449.66	486.55 ± 553.37	0.430
Substance P	7292.64 ± 3021.99	9400.24 ± 3951.42	0.021
Low-sensitivity group (pg/mL)			
CGRP	743.21 ± 947.43	674.85 ± 747.57	0.669
NGF	637.06 ± 259.10	724.09 ± 570.66	0.473
NPY	552.58 ± 524.42	627.52 ± 595.59	0.169
VIP	7957.39 ± 3744.23	9103.56 ± 4619.24	0.169
Substance P	3491.43 ± 2170.61	4986.70 ± 4306.55	0.071

CGRP, calcitonin gene-related peptide; NGF, nerve growth factor; NPY, neuropeptide Y; VIP, vasoactive intestinal peptide.

## Data Availability

The datasets used and analyzed during the current study are available from the corresponding author on reasonable request.
